# Novel putative interactors of FZO-1/mitofusin 2 identified using large-scale yeast two-hybrid screening in *C. elegans*

**DOI:** 10.17912/micropub.biology.000674

**Published:** 2022-12-01

**Authors:** Samiksha Dhananjay, Gursimran Chandhok, Brent Neumann

**Affiliations:** 1 Neuroscience Program, Biomedicine Discovery Institute and Department of Anatomy and Developmental Biology, Monash University, Melbourne VIC 3800 Australia.

## Abstract

Mitochondria are energy-converting organelles that shift between fusion and fission states in order to perform a variety of essential functions. Disruption of these dynamics is detrimental to cellular health and is associated with a range of human diseases. Mitofusin 2 is an essential large GTPase protein that orchestrates fusion of outer mitochondria membranes, and mutations in the encoding gene are causative for Charcot-Marie-Tooth disease. In order to gain further insights into the function of this crucial protein, we have performed large-scale yeast two-hybrid screening to identify interactors of the orthologous protein in *Caenorhabditis elegans* (FZO-1) *.* From this screening, we identified 12 novel interactors of FZO-1/mitofusin 2 that, based on their known functions, are strong candidates for further study.

**Figure 1. Identification of FZO-1 interacting proteins:**
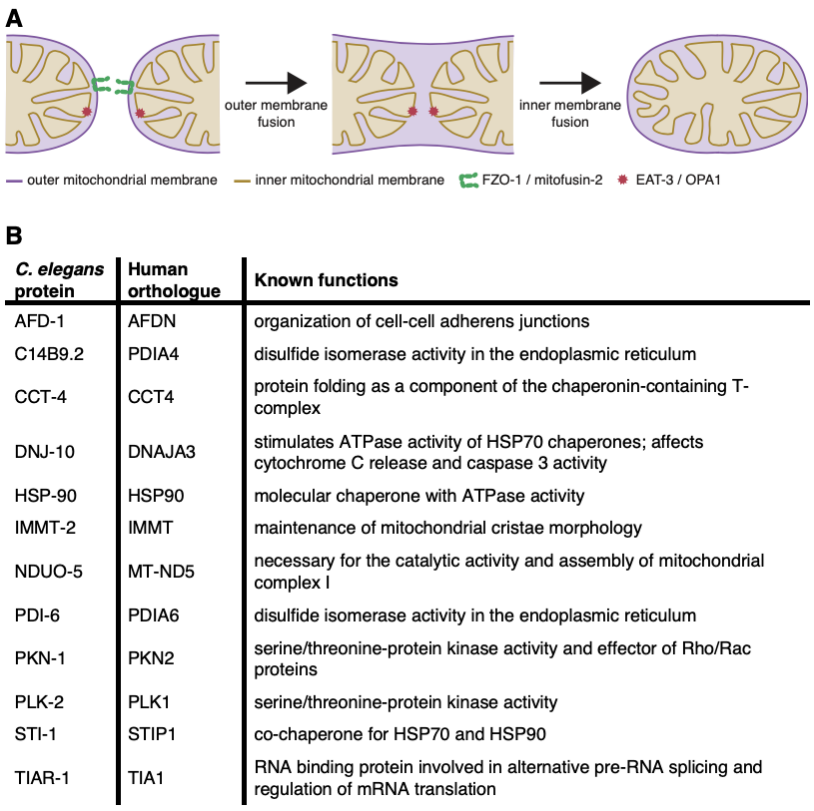
( **A** ) Schematic provides an overview of the mitochondrial fusion process carried out by FZO-1/mitofusin 2 and EAT-3/OPA1. ( **B** ) The proteins identified to interact with FZO-1 by yeast two-hybrid screening are shown together with their human orthologues and known functions.

## Description

Mitochondria are dynamic organelles with a unique ability to transition between opposing fusion and fission states, resulting in changes in their morphology and movement, and allowing them to exist in large interconnected networks. This ability to shift between fusion/fission states allows mitochondria to adapt to changing cellular conditions and maintain their essential functions in meeting cellular bioenergetic demands, regulating intracellular Ca ^2+^ , and mediating the cellular stress response (Chandhok et al. 2018). The fusion process allows material to be exchanged between mitochondria, providing a method for damaged mitochondria to regain essential components and thus mitigate stress (Ono et al. 2001). In contrast, fission largely serves as a quality control measure by allowing deteriorated components of damaged mitochondria to be budded off for targeted breakdown via autophagy or mitophagy (Westermann 2012). Thus, dysfunctional mitochondrial dynamics can have detrimental consequences for organelle function and are intricately linked to cellular health (Yapa et al. 2021).

Mitochondrial fusion is a complex process whereby the outer and inner mitochondrial membranes of two mitochondria merge into one. In humans, mitofusin 1 and 2 (MFN1/2) on the outer mitochondrial membrane function together with optic atrophy 1 (OPA1) on the inner mitochondrial membrane to orchestrate mitochondrial fusion (Westermann 2010) (Figure 1A). MFN1/2 possess several conserved domains: a GTPase domain required for hydrolysis of guanosine triphosphate (GTP) to guanosine diphosphate (GDP); two heptad repeat regions important for the tethering and fusion of adjacent mitochondrial membranes; and transmembrane domains that anchor the protein in the outer mitochondrial membrane. These large dynamin-like GTPases form dimeric interactions, tethering adjacent mitochondria and use GTP hydrolysis to induce fusion (Fritz et al. 2001). Furthermore, MFN2 is associated with several other important cellular functions, including the tethering of mitochondria to endoplasmic reticulum (ER) membranes, and in the modulation of ER stress signalling (Chandhok et al. 2018). Mutations in MFN2 are causative for Charcot-Marie-Tooth disease (Züchner et al. 2004) and changes in *MFN2* expression are associated with a range of other disorders (Chandhok et al. 2018).

Despite its central role in mitochondrial function, only a few other proteins have been shown to interact with MFN2. Thus, to gain further insights into the function of this crucial protein, we used the N-terminal portion (amino acids 1-617, containing both GTPase and heptad repeat 1 domains) of the *C. elegans* FZO-1 protein as bait for yeast two-hybrid screening. We engaged Hybrigenics Services to conduct a large-scale screen, which evaluated the potential interaction between FZO-1 and a total of 264 million peptides generated from mixed stage *C. elegans* grown under normal or stressed conditions. This screen identified 31 putative interacting proteins (see methods), and we considered 12 of these as strong candidates for further study based on their known functions (Figure 1B). Our screen also identified FZO-1 itself, which represents an important positive control given that FZO-1/MFN2 is known to dimerize.

The best characterized functions for each of the putative interacting proteins is listed in Figure 1B. Although MFN2 localizes to the outer mitochondrial membrane, two of the identified proteins (IMMT-2 and NDUO-5) are specifically associated with the inner mitochondrial membrane. IMMT-2/IMMT is crucial for the maintenance of cristae formation (Head et al. 2011), while NDU-5/MT-ND5 is a core component of complex I of the mitochondrial electron transport chain (Bourges et al. 2004). Interestingly, we recently reported severe structural defects in mitochondrial cristae in *C. elegans* muscles lacking FZO-1 (Byrne et al. 2019). Thus, interaction between FZO-1 and proteins linked to the inner mitochondrial membrane may offer insights into how cristae morphology is disrupted in the absence of FZO-1.

Two of the putative FZO-1/MFN2 interactors, the heat shock (HS) chaperone proteins HSP-90 and STI-1, were previously predicted to physically interact with one another (Zhong and Sternberg 2006). HSP-90/HSP90 is a molecular chaperone important for the folding and maturation of a large number of client proteins, and functions together with co-chaperones including STI-1/STIP1 (Lackie et al. 2017). DNJ-10/DNAJA3 is a heat shock mitochondrial chaperone that activates HSP70, a major partner of HSP90 (Craig and Marszalek 2017). In addition, DNJ-10 has been implicated in mitochondrial dynamics along with the other *C. elegans* orthologues of HS proteins (Kirstein-Miles and Morimoto 2010; Li et al. 2009). It is possible that these chaperones interact with FZO-1 to ensure that it is folded correctly following translation, and may therefore be required for its function.

Three additional proteins have previously been shown to physically interact with the *C. elegans* FZO-1 protein (CED-9 (Rolland et al. 2009), IGDB-2 (Wang et al. 2017), and LIN-53 (Müthel et al. 2019)), none of which were identified in our screen. Rolland et al. found that CED-9 interacts predominantly with the central domain (amino acids 346-595) of FZO-1. These authors found that a truncated version of FZO-1 that was highly similar to that used in our current screen (amino acids 1-618 vs. 1-617) displayed a low affinity interaction with CED-9, which they proposed was caused by an inhibitory domain located between amino acids 220–345 (Rolland et al. 2009). This may explain why we did not identify CED-9 in our screen. FZO-1 was found to interact with IGDB-2 and LIN-53 from large-scale mass spectrometry screens (Müthel et al. 2019; Wang et al. 2017). It is possible that these proteins were not identified in our screen because they interact with the C-terminal region of FZO-1 that was not included in our bait peptide or that they were not captured using our different screening methods.

Although the role of MFN2 in mitochondrial dynamics is well established, the molecular and genetic modifiers of MFN2/FZO-1 are not fully understood. As we will not be pursuing this line of research further, we hope that these preliminary finding will be of interest to researchers in the field who will be able to confirm the putative interacting proteins as true MFN2/FZO-1 interactors, and thereby enhance our understanding of this crucial mitochondrial-associated protein.

## Methods

Molecular biology was performed using standard techniques (Sambrook, Fritsch and Maniatis 1989). A full length *fzo-1* cDNA sequence was generated using an ImProm-II Reverse Transcription System (Promega, Wisconsin USA), inserted into BamHI/NheI sites in a *pSM* plasmid backbone, and provided to Hybrigenics Services (Paris, France). Hybrigenics Services subcloned the N-terminal portion of *fzo-1* (encoding amino acids 1-617, containing both the GTPase and heptad repeat 1 domains) into a GAL4 vector for screening.

The truncated FZO-1 protein was used as bait for yeast two-hybrid screening, with 264 million potential interactions examined from *C. elegans* peptide libraries. Libraries were produced and analysed by Hybrigenics Services by pooling polyA+ RNA from three different N2 *C. elegans* cultures. Two of these contained 50% hermaphrodites and males at all developmental stages grown under normal (20°C) or stressed conditions (either starved for 7 h or heat-shocked at 35°C for 1 h prior to a 6 h recovery at 20°C). The third consisted of dauer larvae produced by growth in liquid culture at 22°C to 25°C until dauer arrest occurred. Following screening, Hybrigenics Services provided a list of putative interactors identified.

A total of 31 putative interactors were identified: AFD-1, ALR-1, C14B9.2, C35D10.12, CCT-4, CTC-2, DNJ-10, EIF-3.G, F08F8.9 isoform b, F22D6.2, F28C1.1, FZO-1, HSP-90, IMMT-2, K04C2.2, K04G7.1, LIN-22, LIR-3, NDUO-1, NDUO-5, PDI-6, PKN-1, PLK-2, STI-1, SUCO-1, T05A12.3, TIAR-1, UNC-22, USP-5, WRT-2, Y59A8B.10 isoform a.

## References

[R1] Bourges I, Ramus C, Mousson de Camaret B, Beugnot R, Remacle C, Cardol P, Hofhaus G, Issartel JP (2004). Structural organization of mitochondrial human complex I: role of the ND4 and ND5 mitochondria-encoded subunits and interaction with prohibitin.. Biochem J.

[R2] Byrne JJ, Soh MS, Chandhok G, Vijayaraghavan T, Teoh JS, Crawford S, Cobham AE, Yapa NMB, Mirth CK, Neumann B (2019). Disruption of mitochondrial dynamics affects behaviour and lifespan in Caenorhabditis elegans.. Cell Mol Life Sci.

[R3] Chandhok G, Lazarou M, Neumann B (2017). Structure, function, and regulation of mitofusin-2 in health and disease.. Biol Rev Camb Philos Soc.

[R4] Craig EA, Marszalek J (2017). How Do J-Proteins Get Hsp70 to Do So Many Different Things?. Trends Biochem Sci.

[R5] Fritz S, Rapaport D, Klanner E, Neupert W, Westermann B (2001). Connection of the mitochondrial outer and inner membranes by Fzo1 is critical for organellar fusion.. J Cell Biol.

[R6] Head BP, Zulaika M, Ryazantsev S, van der Bliek AM (2011). A novel mitochondrial outer membrane protein, MOMA-1, that affects cristae morphology in Caenorhabditis elegans.. Mol Biol Cell.

[R7] Kirstein-Miles J, Morimoto RI (2010). Caenorhabditis elegans as a model system to study intercompartmental proteostasis: Interrelation of mitochondrial function, longevity, and neurodegenerative diseases.. Dev Dyn.

[R8] Lackie RE, Maciejewski A, Ostapchenko VG, Marques-Lopes J, Choy WY, Duennwald ML, Prado VF, Prado MAM (2017). The Hsp70/Hsp90 Chaperone Machinery in Neurodegenerative Diseases.. Front Neurosci.

[R9] Li J, Cai T, Wu P, Cui Z, Chen X, Hou J, Xie Z, Xue P, Shi L, Liu P, Yates JR 3rd, Yang F (2009). Proteomic analysis of mitochondria from Caenorhabditis elegans.. Proteomics.

[R10] Müthel S, Uyar B, He M, Krause A, Vitrinel B, Bulut S, Vasiljevic D, Marchal I, Kempa S, Akalin A, Tursun B (2019). The conserved histone chaperone LIN-53 is required for normal lifespan and maintenance of muscle integrity in Caenorhabditis elegans.. Aging Cell.

[R11] Ono T, Isobe K, Nakada K, Hayashi JI (2001). Human cells are protected from mitochondrial dysfunction by complementation of DNA products in fused mitochondria.. Nat Genet.

[R12] Rolland SG, Lu Y, David CN, Conradt B (2009). The BCL-2-like protein CED-9 of C. elegans promotes FZO-1/Mfn1,2- and EAT-3/Opa1-dependent mitochondrial fusion.. J Cell Biol.

[R13] Sambrook J, Fritsch EF, Maniatis T. 1989. *Molecular cloning: a laboratory manual* : Cold spring harbor laboratory press.

[R14] Wang W, Perens EA, Oikonomou G, Wallace SW, Lu Y, Shaham S (2017). IGDB-2, an Ig/FNIII protein, binds the ion channel LGC-34 and controls sensory compartment morphogenesis in C. elegans.. Dev Biol.

[R15] Westermann B (2010). Mitochondrial fusion and fission in cell life and death.. Nat Rev Mol Cell Biol.

[R16] Westermann B (2012). Bioenergetic role of mitochondrial fusion and fission.. Biochim Biophys Acta.

[R17] Yapa NMB, Lisnyak V, Reljic B, Ryan MT (2021). Mitochondrial dynamics in health and disease.. FEBS Lett.

[R18] Zhong W, Sternberg PW (2006). Genome-wide prediction of C. elegans genetic interactions.. Science.

[R19] Züchner S, Mersiyanova IV, Muglia M, Bissar-Tadmouri N, Rochelle J, Dadali EL, Zappia M, Nelis E, Patitucci A, Senderek J, Parman Y, Evgrafov O, Jonghe PD, Takahashi Y, Tsuji S, Pericak-Vance MA, Quattrone A, Battaloglu E, Polyakov AV, Timmerman V, Schröder JM, Vance JM (2004). Mutations in the mitochondrial GTPase mitofusin 2 cause Charcot-Marie-Tooth neuropathy type 2A.. Nat Genet.

