# Erratum: Novel putative interactors of FZO-1/mitofusin 2 identified using large-scale yeast two-hybrid screening in *C. elegans*

**DOI:** 10.17912/micropub.biology.000728

**Published:** 2022-12-22

## Description

A version of the article with incorrect author order was uploaded to PubMedCentral, and subsequently to PubMed. 

The corrected article has author order Dhananjay S, Chandhok G., Neumann B., and citation:

Dhananjay, S; Chandhok, G; Neumann, B (2022). Novel putative interactors of FZO-1/mitofusin 2 identified using large-scale yeast two-hybrid screening in C. elegans. microPublication Biology. 10.17912/micropub.biology.000674. PMID: 36530473; PMCID: PMC9756088. 

